# Estimating Clinically Relevant Cut-Off Values for a High-Throughput Quantitative Real-Time PCR Detecting Bacterial Respiratory Pathogens in Cattle

**DOI:** 10.3389/fvets.2021.674771

**Published:** 2021-05-25

**Authors:** Alicia F. Klompmaker, Maria Brydensholt, Anne Marie Michelsen, Matthew J. Denwood, Carsten T. Kirkeby, Lars Erik Larsen, Nicole B. Goecke, Nina D. Otten, Liza R. Nielsen

**Affiliations:** ^1^Department of Veterinary and Animal Sciences, Faculty of Health and Medical Sciences, University of Copenhagen, Copenhagen, Denmark; ^2^Centre for Diagnostics, Technical University of Denmark, Kongens Lyngby, Denmark

**Keywords:** bovine respiratory disease, calf, diagnostics, nasal swab, rtPCR, clinically relevant cut-off

## Abstract

Bovine respiratory disease (BRD) results from interactions between pathogens, environmental stressors, and host factors. Obtaining a diagnosis of the causal pathogens is challenging but the use of high-throughput real-time PCR (rtPCR) may help target preventive and therapeutic interventions. The aim of this study was to improve the interpretation of rtPCR results by analysing their associations with clinical observations. The objective was to develop and illustrate a field-data driven statistical method to guide the selection of relevant quantification cycle cut-off values for pathogens associated with BRD for the high-throughput rtPCR system “Fluidigm BioMark HD” based on nasal swabs from calves. We used data from 36 herds enrolled in a Danish field study where 340 calves within pre-determined age-groups were subject to clinical examination and nasal swabs up to four times. The samples were analysed with the rtPCR system. Each of the 1,025 observation units were classified as sick with BRD or healthy, based on clinical scores. The optimal rtPCR results to predict BRD were investigated for *Pasteurella multocida, Mycoplasma bovis, Histophilus somni, Mannheimia haemolytica*, and *Trueperella pyogenes* by interpreting scatterplots and results of mixed effects logistic regression models. The clinically relevant rtPCR cut-off suggested for *P. multocida* and *M. bovis* was ≤ 21.3. For *H. somni* it was ≤ 17.4, while no cut-off could be determined for *M. haemolytica* and *T. pyogenes*. The demonstrated approach can provide objective support in the choice of clinically relevant cut-offs. However, for robust performance of the regression model sufficient amounts of suitable data are required.

## Introduction

Bovine respiratory disease (BRD) is a multifactorial disease which involves multiple stressors, environmental and host factors, and various infectious agents. The disease is a common health problem and a cause of mortality and welfare issues in calves between 1 and 6 months old (1, 2). Furthermore, BRD is associated with economic losses due to direct costs of treatment and lost calves, but also because of long-term impacts on animal performance e.g., reduced weight gain and thereby age at first calving (3). Costs of treatment for respiratory disease in feedlot cattle in the United States were estimated to ~€20 per case (4), while a Dutch study estimated annual losses per dairy heifer to €31 on average (5). Respiratory disease among calves is also associated with high levels of antimicrobial use. In the year 2015, antibiotics registered for respiratory disease covered 71% of the total amount of antibiotics prescribed for Danish calves (6).

Obtaining a timely and accurate diagnosis of BRD is challenging (7) due to the uncertainty of whether pathogens recovered from the sample are in fact the cause of the respiratory disease or simply a part of the microbiota. Ante-mortem diagnosis of respiratory disease is typically based on clinical examinations (8), where the most common clinical signs are fever, coughing, nasal- and ocular discharge, depression, increased respiratory rate and laboured breathing (9). Clinical respiratory scoring systems to detect calves with respiratory disease have been developed and scientifically validated with moderate sensitivity and relatively high specificity (10). However, the clinically sick animal will rarely display signs which are specific for a single aetiology (7). Diagnostic laboratory testing is therefore necessary for identification of the pathogens associated with BRD (11), allowing correct treatment and prevention to be initiated. A new high-throughput real-time PCR (rtPCR) detection system using the BioMark HD platform (Fluidigm, South San Francisco, USA) established at the Centre for Diagnostics, Technical University of Denmark can detect genetic material from multiple bovine viruses and bacteria in the same setup while running numerous samples at once.

We used this new high-throughput rtPCR system for detection of nine respiratory agents. In this manuscript, we report on analysis of five of these for which we had a presumably sufficient sample of test-positive samples, namely *Mycoplasma bovis* (*M. bovis*), *Histophilus somni* (*H. somni*), *Mannheimia haemolytica* (*M. haemolytica*), *Pasteurella multocida* (*P. multocida*), and *Trueperella pyogenes* (*T. pyogenes*) in nasal swabs. Several studies in the veterinary and human medical field have shown that clinical presentation and disease severity can be related to (semi-)quantitative PCR results representing the pathogenic load (12–14). On the other hand, several pathogens associated with BRD can also be found in clinically healthy animals showing that merely detecting the pathogen is not sufficient for making a diagnosis (15). To the authors' knowledge, clinically relevant rtPCR cut-off values have not yet been defined for bovine respiratory pathogens. Therefore, there is a need to determine cut-offs for which the test result is associated with respiratory disease and not just presence of the pathogen, thereby improving the interpretation of molecular diagnostics, to assist veterinarians and farmers in making more objective and accurate interventions. The study objective was to develop and provide proof-of-concept of a new data-driven statistical approach, providing evidence to suggest field-relevant rtPCR quantification cycle (Cq) cut-off values, and to test this model on common bovine pathogens associated with respiratory disease tested by the high-throughput rtPCR system (Fluidigm). The study was based on data from a Danish field study providing systematically collected clinical recordings paired with rtPCR results from nasal swab samples.

## Materials and Methods

### Herd and Calf Selection

The data used in this study were collected between September 2018 and March 2020. A total of 36 cattle herds including nine veal herds and 27 dairy herds participated. In Denmark, a veal herd is a rosé veal calf producing unit, where mainly bull calves purchased from dairy herds are slaughtered as veal (8–12 months) or young bulls (>12 months) (16). Selection criteria for veal herds were the use of electronic disease registration and regular dairy calf suppliers. Qualifying herds were selected by convenience to ensure wide geographical coverage in Denmark. For each of the nine selected veal herds, the three dairy farms supplying the highest number of calves on a regular basis were asked to participate. For all 36 herds, participation was voluntary.

At the beginning of the study period for each herd, up to 12 calves between 0 and 10 days old were randomly selected (this age group is referred to as “Age 1w”). On most farms, 12 calves were not yet available for sampling at the first herd visit, so follow-up visits were necessary to increase the number of animals. The lack of calves at initial visits also meant that it was not always possible to select calves at random, but necessary to include all available calves. The selected cohorts of calves were subsequently examined at 3 weeks of age (“Age 3w”), 2 weeks after introduction to the veal herds (“Age 2wai”), and at 3 months of age (“Age 3m”), resulting in four age groups. A total of 340 individual calves were sampled up to four times resulting in 1,025 observation units. In this study, an observation unit refers to a calf in a particular age group.

### Clinical Examination and Sample Collection

A clinical examination protocol was developed prior to herd visits, and the participating veterinary researchers underwent a joint training session aiming to harmonise their scoring. Clinical examinations were primarily performed by two veterinarians, and data were registered onsite and synchronised with an online platform and project database. Clinical measures relevant to the study presented in this paper included rectal temperature and coughing as well as nasal and ocular discharge. Each calf was also subject to nasal swab collection using 15 cm unguarded polyester-tipped swabs. A swab was guided into one naris, rotated against the mucosal wall, and withdrawn. The tip of the swab was placed in an Eppendorf tube containing PBS and stored at ~5°C for a maximum of 4 days until sample preparation and extraction.

### Sample Analysis

The nasal swab samples were analysed at the Centre for Diagnostics, Technical University of Denmark. Samples were vortexed, after which bacterial DNA were extracted using the extraction robot QIAcube HT (QIAGEN, Hilden, Germany) and the Cador Pathogen 96 QIAcube HT kit (QIAGEN) according to the manufacturer's instructions. The extracted samples were pre-amplified (DNA-targets) as described by Goecke et al. (17). This pre-amplification step prior to the rtPCR, results in distinctly lower Cq values compared to other rtPCR systems. The primer and probe sequences used were either from previously published assays or designed for the project (17). For the high-throughput rtPCR amplification, the BioMark HD (Fluidigm) and the BioMark 192.24 Dynamic Array (DA) Integrated Fluidic Circuit (IFC) chip (Fluidigm) were used. This platform automatically combines 192 pre-amplified samples with 24 assays, thus enabling 4,608 individual rtPCR reactions simultaneously. The chip was placed in the RX IFC controller (Fluidigm) for loading and mixing. After 30 min, the chip was transferred to and run on the rtPCR BioMark HD platform. Known positive and negative control samples were included in each run, and if the Cq values of the positive controls were not within two Cq values of the pre-determined value, the DA IFC chip was run again. The output data, including the Cq values and amplification curves, obtained from the BioMark HD system were analysed using the Fluidigm Real-Time PCR Analysis software 4.1.3 (Fluidigm).

### Respiratory Clinical Status

The calves were classified as either sick with respiratory disease or not, using a clinical scoring system. Calves classified as not having respiratory disease are referred to as healthy for the purpose of this study. The scoring system was adapted from two existing scoring systems for BRD (11, 18). The clinical score was based on the four clinical signs: coughing, rectal temperature, nasal discharge, and ocular discharge, where both unilateral and bilateral discharge were considered on equal terms. For each sign, points were given depending on the severity, and the total of these points for each sign equalled the clinical score for the calf. For all signs, a score of zero was given if the sign was not present, or in the case of rectal temperature, if the temperature was <39° C. For nasal discharge, serous discharge equalled one point while mucopurulent discharge equalled four points. For ocular discharge, serous discharge equalled one point while mucopurulent discharge equalled two points. In addition, one point was added to the total score if a calf had both nasal and ocular discharge of any severity. For rectal temperature, 39–39.3°C equalled one point while a temperature ≥ 39.4°C equalled two points. Finally, at least one spontaneous cough equalled three points. A calf with a clinical score equal to or greater than five points was classified as sick with respiratory disease.

### Statistical Analyses

Statistical analyses were conducted using the statistical software R (19). All data were extracted from the SQL database located at Aarhus University into an Excel spreadsheet pairing clinical registrations and rtPCR Cq values for each observation unit. The pathogens for which attempts were made to find a clinically relevant Cq value were *M. bovis, H. somni, M. haemolytica, P. multocida*, and *T. pyogenes*.

Scatterplots depicting the clinical score for each observation unit plotted against Cq values were created for each of the five tested pathogens. The scatterplots were visually inspected to attempt to identify a plausible Cq cut-off value for each pathogen by considering the distribution of Cq values for observation units defined as either sick with respiratory disease or not. To aid the visualisation of a cut-off, data points were displayed as black dots if the observation units were classified as sick with respiratory disease, or grey triangles if not. Nasal swab samples in which the pathogen in question was not found were given the Cq value 32 (referred to as test-negative), as no samples with a Cq value (referred to as test-positive) reached this value. Both positive and negative test-results were included in the scatter plots.

The following procedure was used to determine the “optimal cut-off value,” i.e., the cut-off value leading to the test-result interpretation with the highest predictive value for respiratory disease being present in the calves. First, an interval of Cq values in which to search for optimal cut-off values was specified for each pathogen. The interval was based on the observed distribution of Cq values and excluded the extreme ends of the distribution with sparse data. Each of the potential cut-off values for rtPCR Cq within this interval were then evaluated based on the predictive ability to determine the observation units' respiratory health status using a mixed-effects logistic regression model fit using the lme4 package (20) within R (19). The outcome variable “sick with respiratory disease” was dichotomised into yes or no, with yes referring to observation units with a clinical score equal to or above five, and no referring to observation units with a clinical score below five. The main explanatory variable of interest was the rtPCR results dichotomised according to the threshold being tested. The explanatory variable “Age Group” had four levels as explained above. Data hierarchies (e.g., data with a nested or clustered structure) were adjusted for in the mixed-effects model at herd- and age-group level by including them as a combined random effect factor (“Group ID”). The model was re-fit using each potential value of rtPCR threshold. Predicted probability plots were then created for each pathogen based on the predictive ability of each mixed-effects model.

## Results

### Descriptive Analyses

A total of 1,025 observations were available from 340 calves. In each of the nine veal herds between 14 and 36 individual calves were sampled. In the 27 dairy herds between three and 33 individual calves were sampled in each herd. In total, 417 (40.7%) observation units were scored as being sick with respiratory disease (Age 1w: 17.5%, Age 3w: 9.4%, Age 2wai: 29.3%, Age 3m: 43.9%). Hence, the prevalence of BRD was generally higher in older than younger calves. This was taken into account in the model results reported below.

Of these, 296 (71%) were positive (had DNA-material detected as defined by Cq <32) for at least one of the five investigated respiratory pathogens, and 121 (29%) were test-negative. The median clinical score and mean age of the sick observation units were seven points and 57 days (SD 35.7), respectively. The remaining 608 (59.3%) observation units had a clinical score below five and were per definition scored as not being sick with respiratory disease. In 362 (59.5%) of these 608 observation units none of the pathogens were detected, while in 246 (40.5%) observation units, DNA from at least one pathogen was detected. The median clinical score and mean age for the observation units classified as healthy were two points and 29 days (SD 31.5), respectively.

The data for the five investigated pathogens are summarised in Table 1. Generally, the minimum and maximum Cq values for these pathogens did not differ meaningfully. For observation units, which had measurable Cq values for *M. bovis, H. somni, M. haemolytica*, and *P. multocida*, ~60–70% were classified as sick with respiratory disease. However, only 36.6% of the observation units with Cq values <32 for *T. pyogenes* were classified as sick. The mean age for observation units positive for *T. pyogenes* was lower for both healthy and sick calves, compared to the other four pathogens. *H. somni* was the pathogen detected the fewest times as the only pathogen detected in a sample. The pathogens were most often found in combination with *P. multocida*.

**Table 1:** Summary of the pathogens [*M. bovis* (MB), *H. somni* (HS), *M. haemolytica* (MH), *P. multocida* (PM), and *T. pyogenes* (TP)] with the percentage of the observation units, in which the respective pathogen was detected, which were classified as sick and the minimum and maximum Cq value for both healthy (H) and sick (S) observation units.

		**Cq value**										
		**Min**		**Max**		**Median score**		**Mean age**		**# detected as the only pathogen**		
**Pathogen**	**Scored as sick (%)**	**H**	**S**	**H**	**S**	**H**	**S**	**H**	**S**	**H**	**S**	**In combination with (%)**
MB	64.8	13.1	12.8	27.5	27.8	4	7	44.5	55.0	17	19	PM (56) MH (37)
HS	69.4	15.6	11.9	24.8	25.1	4	7	75.8	85.8	3	7	PM (68) MH (47)
MH	60.0	10.5	11.1	26.7	24.4	3	7	65.0	74.9	23	17	PM (56) MB (30)
PM	61.0	12.5	12.9	25.2	26.6	3	7	61.1	70.9	51	76	MH (30) MB (25)
TP	36.6	13.8	17.8	26.3	25.1	2	7	23.5	47.6	66	22	PM (31) MH (19)

*The median clinical score and mean age for the healthy (H) and sick (S) observation units and the number of healthy (H) and sick (S) observation units where the pathogen was detected as the only pathogen. Finally, the pathogens which the respective pathogen was most often found in combination with are shown*.

### Real-Time PCR Cut-Off Analyses

#### P. multocida

**Figure 1:**
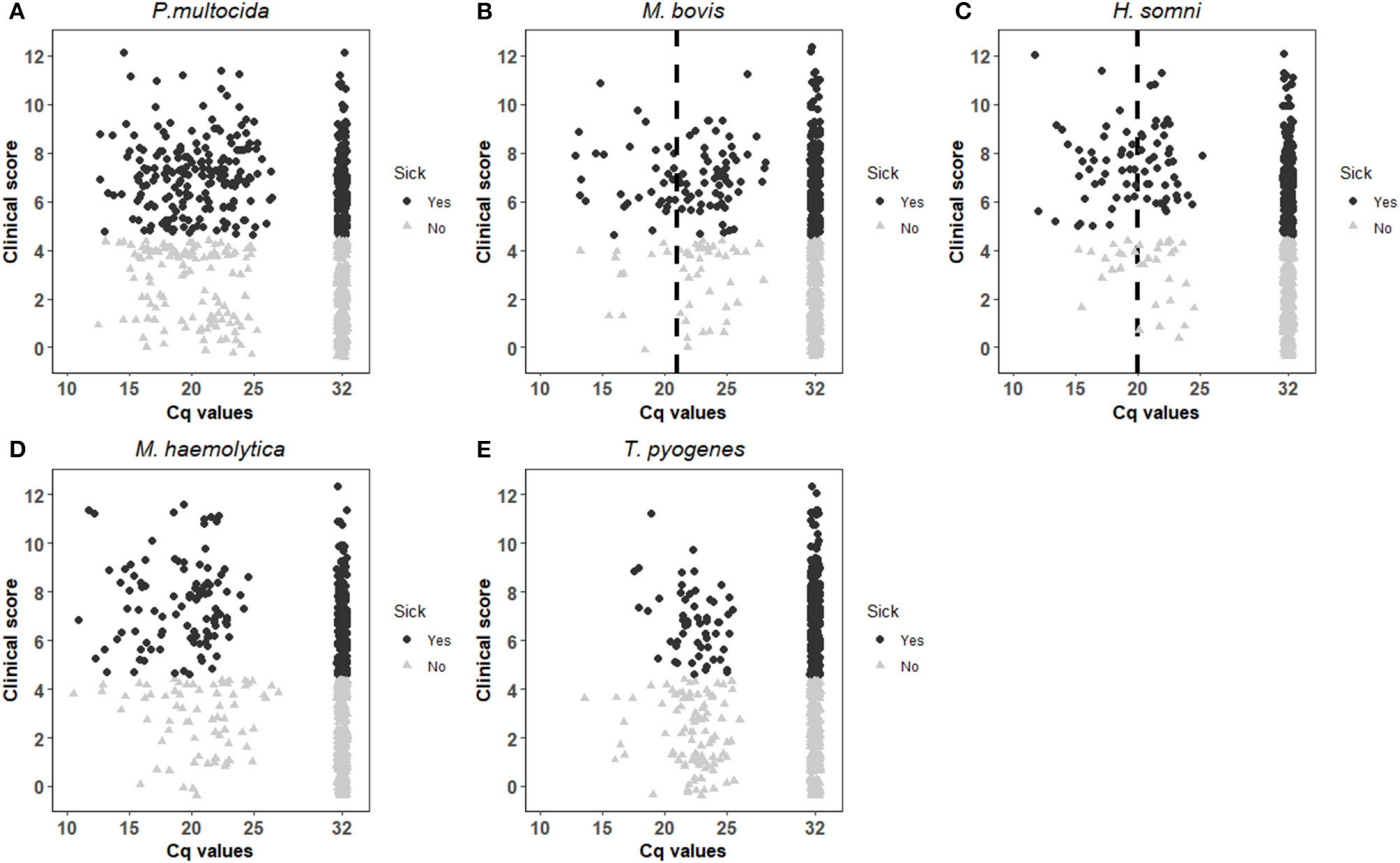
Distribution of respiratory clinical score against Cq values for *P. multocida* **(A)**, *M. bovis* **(B)**, *H. somni* **(C)**, *M. haemolytica* **(D)** and *T. pyogenes* **(E)**, and additionally grouped by respiratory disease classification with black dots indicating an observation unit sick with respiratory disease and grey triangles indicating a healthy. The vertical dotted lines in **(B,C)** represent the visually estimated clinically relevant Cq values.

**Figure 2:**
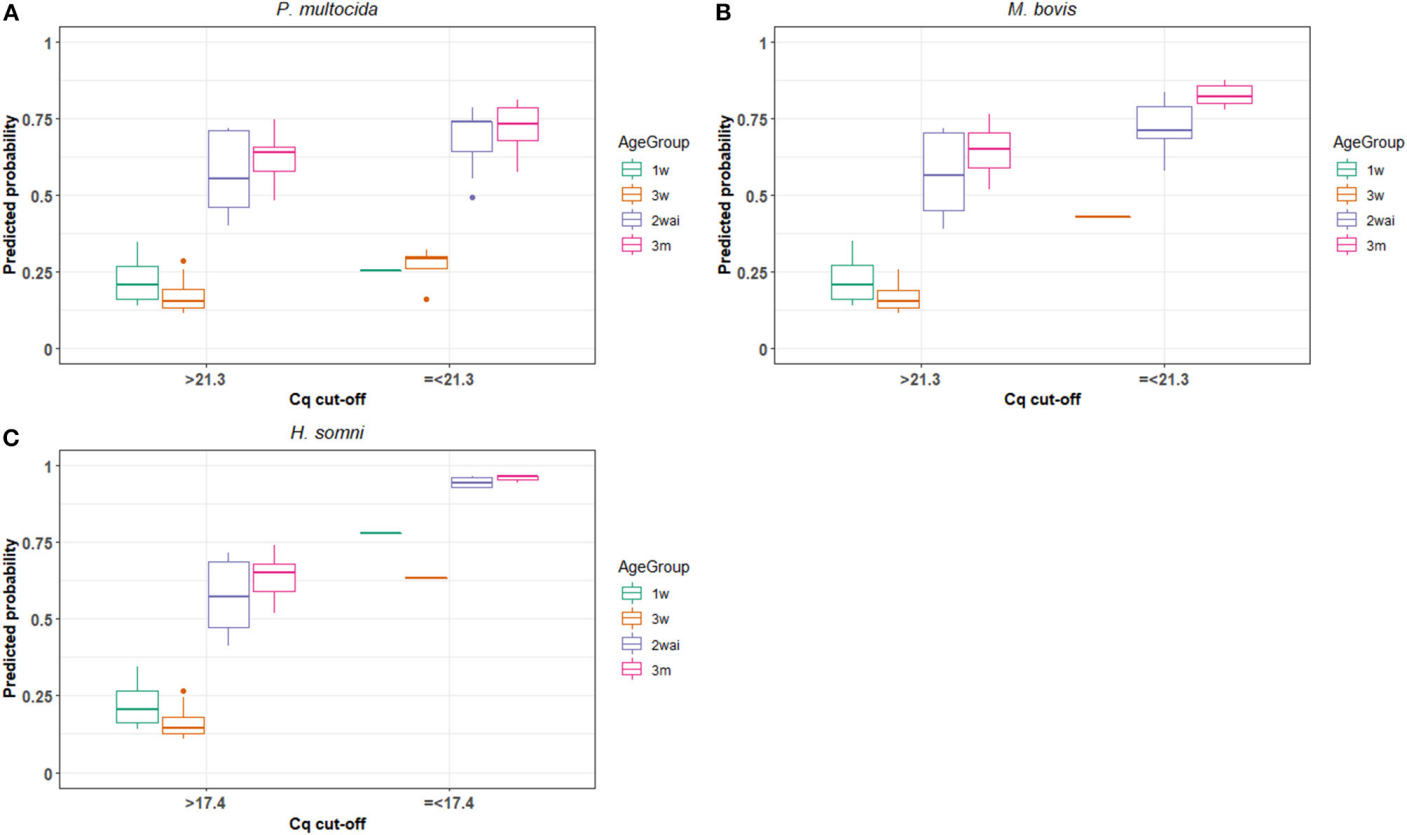
The model predicted probability of calves being classified as sick with respiratory disease using the Cq cut-offs providing the highest predictive value of the Fluidigm rt-PCR for detection of *P. multocida* **(A)**, *M. bovis* **(B)** and *H. somni* **(C)** in each age group of calves in 27 dairy and nine veal herds (1w, 1 week old; 3w, 3 weeks old; 2wai, 2 weeks after introduction to veal herds; and 3m, 3 months old).

Based on the scatterplot in Figure 1, it was not possible to visually estimate a clinically relevant cut-off for *P. multocida*, because it did not show any clear association between clinical score and the rtPCR results. The interval chosen for the mixed-effects model for *P. multocida* was 14–25 and the results are given in Table 2. Dichotomised rtPCR results based on a cut-off of Cq ≤ 21.3 was found to be significantly associated with being scored as sick with respiratory disease (*p* = 0.026). The proportion of observation units with *P. multocida* Cq values ≤ 21.3 being classified as sick with respiratory disease was 0.61, whereas it was 0.36 for observation units with Cq values > 21.3. In Figure 2, the predicted probability plot illustrates that the probability of being classified as sick with respiratory disease within each age-group for samples with *P. multocida* Cq ≤ 21.3 was slightly higher than when the Cq was >21.3, and the difference was largest in the 3w age-group.

**Table 2:** Results of the mixed-effects model for analysis of variables associated with respiratory disease in calves including analysis of the best rtPCR Cq cut-off differentiating between sick and healthy calves for *P. multocida, M. bovis, H. somni, M. haemolytica* and *T. pyogenes*.

**Pathogen**	**Variables**	**Estimate**	**SE**	***p***	**σ^2^**	**SD**
*P. multocida*	*Fixed effects*					
	Intercept	−1.33	0.19	***		
	Cq > 21.3	0	–	–		
	Cq ≤ 21.3	0.44	0.2	*		
	Age 1w	0	–	–		
	Age 3w	−0.30	0.29	–		
	Age 2wai	1.66	0.32	***		
	Age 3m	1.85	0.28	***		
	*Random effect*					
	Group ID				0.34	0.58
*M. bovis*	*Fixed effects*					
	Intercept	−1.31	0.19	***		
	Cq > 21.3	0	–	–		
	Cq ≤ 21.3	0.77	0.38	*		
	Age 1w	0	–	–		
	Age 3w	−0.29	0.28	–		
	Age 2wai	1.63	0.31	***		
	Age 3m	1.96	0.27	***		
	*Random effect*					
	Group ID				0.32	0.56
*H. somni*	*Fixed effects*					
	Intercept	−1.33	0.18	***		
	Cq > 17.4	0	–	–		
	Cq ≤ 17.4	2.38	0.75	**		
	Age 1w	0	–	–		
	Age 3w	−0.34	0.28	–		
	Age 2wai	1.75	0.30	***		
	Age 3m	1.95	0.27	***		
	*Random effect*					
	Group ID				0.30	0.54
*M. haemolytica*	*Fixed effects*					
	Intercept	−1.31	0.18	***		
	Cq > 22.2	0	–	–		
	Cq ≤ 22.2	0.37	0.23	–		
	Age 1w	0	–	–		
	Age 3w	−0.28	0.28	–		
	Age 2wai	1.67	0.31	***		
	Age 3m	1.88	0.27	***		
	*Random effect*					
	Group ID				0.31	0.56
*T. pyogenes*	*Fixed effects*					
	Intercept	−1.29	0.19	***		
	Cq > 23.7	0	–	–		
	Cq ≤ 23.7	−0.18	0.24	–		
	Age 1w	0	–	–		
	Age 3w	−0.26	0.29	–		
	Age 2wai	1.78	0.31	***		
	Age 3m	1.98	0.27	***		
	*Random effect*					
	Group ID				0.33	0.58

The estimates describe the log-transformed fixed effects of Cq cut-off and age and standard error (SE) and p-value (p) are provided for the estimates. Variance (σ^2^) and standard deviation (SD) are provided for the random effect, GroupID (*p-value < 0.05, **p-value < 0.01, ***p-value < 0.001).

#### M. bovis

By visual inspection of the scatterplot in Figure 1, the cut-off for *M. bovis* was tentatively placed at Cq value 21. Mixed-effects models for *M. bovis* were run over the interval of Cq 15–26 and the results are shown in Table 2. Dichotomised rtPCR results based on a cut-off of ≤ 21.3 was significantly associated with a clinical score indicating respiratory disease (*p* = 0.042). The overall proportion of observation units being classified as sick with respiratory disease with Cq values ≤ 21.3 was 0.72, whereas it was 0.39 for observation units with *M. bovis* Cq values > 21.3. Figure 2 illustrates that the predicted probability of being classified as sick with respiratory disease within each age-group for samples with *M. bovis* Cq ≤ 21.3 was generally higher than when the Cq was >21.3. However, there were no observation units with Cq values ≤ 21.3 in the 1w age-group.

#### H. somni

The visual inspection of Figure 1 placed the Cq cut-off for *H. somni* at Cq 20. For *H. somni*, the mixed-effects model was run over the interval of Cq 15–25, and the results are seen in Table 2. Dichotomised rtPCR based on a cut-off at Cq ≤ 17.4 was significantly associated with a clinical score indicating respiratory disease (*p* = 0.002). The proportion of observation units being classified as sick with respiratory disease with *H. somni* Cq values ≤ 17.4 was 0.86, whereas it was 0.40 for observation units with Cq values > 17.4. Figure 2 illustrates that the predicted probability of being classified as sick with respiratory disease within each age-group for samples with *H. somni* ≤ 17.4 was generally markedly higher than when the Cq was >17.4. However, few observations were available with Cq values ≤ 17.4 in the 1w and 3w age-groups.

#### *M. haemolytica* and *T. pyogenes*

A visual cut-off could not be placed for neither *M. haemolytica* nor *T. pyogenes*, based on Figure 1 because no clear associations between clinical score and Cq values were found. The interval chosen for the mixed-effects model was 14–26 for *M. haemolytica* and 20–26 for *T. pyogenes*. As shown in the results in Table 2, none of the potential cut-offs were found to yield a dichotomised rtPCR result with a significant association with being classified as sick vs. healthy for these pathogens with the available data.

## Discussion

This study shows that it is possible to suggest clinically relevant cut-off values with statistically significant associations with clinical scores indicating respiratory disease for the three pathogens *P. multocida, M. bovis*, and *H. somni*. A cut-off of Cq ≤ 17.4 was found for *H. somni*, accompanied by the highest probability of being scored as sick with respiratory disease.

Meaningful cut-off values could not be determined for *M. haemolytica* and *T. pyogenes* in this study. For *M. haemolytica*, this might be explained by the assay used for the rtPCR in this study, which did not distinguish between different serotypes. Several serotypes of *M. haemolytica* exist, for instance serotype A1, which is associated with clinical disease, and serotype A2, which occurs as a commensal (21). Basing the statistical calculations also on commensal serotypes complicates determining clinically relevant Cq cut-off values. For further investigations, it would be relevant to only test samples for the pathogenic serotypes of *M. haemolytica*.

For *T. pyogenes*, 36.6% of the observation units test-positive (Cq value <32) for this bacterium were scored as sick with respiratory disease, whereas this was more than 60% for the other four pathogens. Furthermore, in most of the observation units positive for *T. pyogenes*, it was found as the only pathogen. These findings suggest that the majority of the *T. pyogenes* detected in this study were a part of the commensal nasal microbiota, as supported by published literature (22), making it difficult to determine a clinically relevant Cq cut-off for that pathogen.

One thing to consider when using the high-throughput rtPCR platform BioMark is the risk of false negative results, which can occur if a sample is very positive. This results from the additional pre-amplification step that may lead to saturation due to the very high level of templates. In such cases, it will be necessary to dilute the sample and re-test it in the BioMark platform. In general, the optimal range of measurable Cq values in the high-throughput rtPCR platform is ~8–10 cycles lower compared to regular rtPCR cyclers (23) and therefore, the cutoff value will also be lower for rtPCR assays in the high-throughput setup.

It was not possible to investigate combinations of pathogens using the optimisation and modelling approach, which would have required a larger dataset with a higher sample size of the different pathogen combinations. Thus, the clinically relevant Cq cut-offs were calculated without accounting for the central interactions between the respiratory pathogens. BRD often has a polymicrobial aetiology and it is likely that the presence of some pathogens influence the presence and/or growth of others (24), thereby affecting the Cq values. In addition, other pathogens than those investigated in the current study are associated with BRD, for instance bovine respiratory syncytial virus, bovine coronavirus, influenza D virus, and bovine parainfluenzavirus type 3. However, result on the viruses were not included in the statistical analyses as there were either very few positive samples, or because it was not yet possible to test for the pathogen using the high-throughput rtPCR system (Fluidigm) as it was set up for this project. To exploit the full potential of the rtPCR system it would be necessary to determine possible cut-off Cq values for the remaining important respiratory infectious agents. As many of the pathogens involved in BRD are also commensals, their mere presence may not be indicative of disease. Establishing clinically relevant Cq cut-offs would make it possible to differentiate between harmless commensals and disease-causing pathogens under different conditions. Furthermore, repeated testing in the same herd would enable improved understanding of the effect of changing pathogen occurrence in age groups or barn sections over time.

Due to the inclusion of multiple factors, the mixed-effects model was valued as the most precise method to indicate relevant cut-offs. However, as evident from the scatterplots, the correlation between Cq values and clinical scores was not very clear. Hence, the level of noise in the data affected the performance of the regression model and the robustness of the model results. This limitation could be levitated by access to larger datasets with more test-positive samples combined with stringent clinical scoring of the calves. Another method frequently used for test comparison and test performance studies in the literature, receiver operating characteristic (ROC) curves, used by e.g., Loy et al. (25), would likely have encountered similar data noise issues.

Another limitation in this study was that the same calves were included as individual observations up to four times (equal to four age groups). However, using the mixed-effects model, a random factor combining herd- and age-group was included. This factor includes herd differences, but also differences between age groups within the herds, such as calf and colostrum management, housing environment etc., all of which could impact disease as well as the calves' ability to overcome disease.

The course of a disease is dynamic and the pathogens which initiate BRD in a calf are not necessarily the same in the later stages or at post-mortem (24). Thomas et al. (15) described varying carriage rates of *H. somni, P. multocida*, and *M. haemolytica* in healthy beef calves. It showed that not all calves became colonised with the bacteria even though they were placed in the same environment. Furthermore, the carriage rates decreased over time which can be explained by the calves getting immunologically more mature and thereby better at clearing pathogens (15). In the present study, 29% of the observation units which were scored as sick with respiratory disease did not have any respiratory pathogens detected in their nasal swabs. Likewise, in 40.5% of the observation units classified as not sick with respiratory disease, respiratory agents able to act as primary pathogens were detected. While one reason for this could be the detection of commensals, another explanation could be that visits to the same calves, in which samples were taken and clinical assessments were performed were carried out with intervals of at least 2 weeks. Therefore, disease course in individual calves could not be followed over time but should rather be considered as snapshots. This may have led to misclassification of BRD status in some observation units. It is thereby possible that a calf presenting with a low clinical score, but a high microbial load (low Cq value) could present with more severe clinical sign in the days following assessment and sampling. Another explanation for this opposing result could be rtPCR detecting DNA material from non-viable organisms still present in the respiratory tract (15).

BRD is a problem at group level as calves are housed together and sometimes mixed from different herds (26). Therefore, it would be interesting to investigate the potential of the rtPCR system to be used at group level and determine relevant cut-off values for group level testing. In groups, calves are usually in different stages of infection at any given time. Therefore, testing groups might be more representative of which pathogens are associated with disease than individual animal testing, as the latter may be more affected by the mentioned snapshots. Again, repeated testing of groups of calves over time would allow for improved understanding of the effect of changing pathogen profiles. It should be emphasised that treatment decisions cannot be made solely based on the results of this test, but should always be made together with clinical evaluations of the calves.

In conclusion, this study demonstrated how the selection of clinically relevant rtPCR Cq cut-offs could be guided by use of a mixed-effects logistic regression model for three well-known BRD-pathogens. A Cq cut-off of ≤ 21.3 was identified for *P. multocida* and *M. bovis*, while a cut-off of ≤ 17.4 was identified for *H. somni*. Further investigations are warranted to define cut-off values for all relevant respiratory pathogens in bovine calves to make more relevant diagnoses and thereby improve treatment and prevention of BRD.

## Data Availability Statement

The raw data supporting the conclusions of this article will be made available by the authors, without undue reservation.

## Ethics Statement

Ethical review and approval was not required for the animal study because before the initiation of this research project, the Animal Experiment Council under the Danish Veterinary and Food Administration was contacted for ethical approval. It was stated by the council that due to the observational and diagnostic nature of the research further approval was not needed. Written informed consent was obtained from the herd owners for the participation of their animals in this study and allowing the researchers access to data from their herds for research purposes. All procedures in the code for conduct of responsible conduct of research at the University of Copenhagen were adhered to.

## Author Contributions

The study was conceived by LN, LL, AM, AK, and MB. AK and MB analysed data and wrote the manuscript. AM, NO, AK, and MB were responsible for data collection. NG was responsible for sample analysis. MD and CK were responsible for the specification of the mixed-effects logistic regression model and statistical supervision. LN, LL, and AM supervised the study and contributed with major revision of the manuscript. All authors contributed to the article and approved the submitted version.

## Conflict of Interest

The authors declare that the research was conducted in the absence of any commercial or financial relationships that could be construed as a potential conflict of interest.
